# Murine cytomegalovirus degrades MHC class II to colonize the salivary glands

**DOI:** 10.1371/journal.ppat.1006905

**Published:** 2018-02-15

**Authors:** Joseph Yunis, Helen E. Farrell, Kimberley Bruce, Clara Lawler, Stine Sidenius, Orry Wyer, Nicholas Davis-Poynter, Philip G. Stevenson

**Affiliations:** 1 School of Chemistry and Molecular Biosciences, University of Queensland, Brisbane, Australia; 2 Child Health Research Center, University of Queensland, South Brisbane, Australia; University Medical Center, Albert-Ludwigs-University Freiburg, GERMANY

## Abstract

Cytomegaloviruses (CMVs) persistently and systemically infect the myeloid cells of immunocompetent hosts. Persistence implies immune evasion, and CMVs evade CD8^+^ T cells by inhibiting MHC class I-restricted antigen presentation. Myeloid cells can also interact with CD4^+^ T cells via MHC class II (MHC II). Human CMV (HCMV) attacks the MHC II presentation pathway *in vitro*, but what role this evasion might play in host colonization is unknown. We show that Murine CMV (MCMV) down-regulates MHC II via M78, a multi-membrane spanning viral protein that captured MHC II from the cell surface and was necessary although not sufficient for its degradation in low pH endosomes. M78-deficient MCMV down-regulated MHC I but not MHC II. After intranasal inoculation, it showed a severe defect in salivary gland colonization that was associated with increased MHC II expression on infected cells, and was significantly rescued by CD4^+^ T cell loss. Therefore MCMV requires CD4^+^ T cell evasion by M78 to colonize the salivary glands, its main site of long-term shedding.

## Author summary

Human cytomegalovirus is the commonest infectious cause of harm to unborn children. Vaccines have not stopped it establishing chronic, systemic infections. Murine cytomegalovirus (MCMV) provides an accessible model to understand why. We show that MCMV evades CD4^+^ T cells via its M78 protein, and that this helps infection to spread despite the immune response. Thus while CD4^+^ T cells are important for host defence, viral evasion limits their capacity to act alone in controlling infection.

## Introduction

Herpesviruses establish persistent, productive infections, with high prevalence in most populations and significant disease burdens. Vaccine development against Human cytomegalovirus (HCMV) is motivated by a high incidence of congenital infection. However vaccines to date have not prevented viral persistence or transmission. CMVs have extensive arsenals of immune evasion genes, so a key task in developing better vaccines is to understand how viral evasion limits immune defence. This requires animal models.

Murine CMV (MCMV) provides an accessible model for HCMV. Both viruses evade CD8^+^ T cells by attacking MHC class I (MHC I)-restricted antigen presentation. For MCMV this promotes salivary gland (SG) infection [1]. Similar evasion by Rhesus CMV helps it to re-infect immune hosts [2]. HCMV also attacks MHC II-restricted antigen presentation, limiting MHC II induction by interferon-γ (IFNγ) [3] and triggering MHC II degradation via US2 [4], US3 [5] and pp65 [6]. CD4^+^ T cell evasion is implied. Nonetheless clinical deficiencies show that CD4^+^ T cells are a key defence against HCMV [7]. They also help to control MCMV in the salivary glands [8] via an IFNγ-dependent mechanism [9]. Thus, CMV infections feature both CD4^+^ T cell evasion and CD4^+^ T cell-dependent control. How these fit together is unclear.

CD4^+^ T cell evasion through attack on MHC II has a less clear rationale than for CD8^+^ T cells and MHC I. MHC II can present cell-endogenous antigens after autophagy [10], but presents mainly cell-exogenous antigens that are recovered from endosomes. Thus the presenting cell need not be infected, making cytotoxicity an inappropriate response to antigen recognition. Moreover, while CD4^+^ T cells can induce target cell apoptosis [11] they are much less effective killers than CD8^+^ T cells. Mainly they activate antigen^+^ presenting cells. γ-herpesviruses even exploit CD4^+^ T cell recognition to drive infected B cell proliferation [12]. CD4^+^ T cell evasion by CMVs suggests that the delivery of activation signals to infected presenting cells would hinder a key aspect of host colonization.

Herpesviruses infect multiple cell types, and the outcome when immunity meets viral evasion can be cell type-dependent. For example CD8^+^ T cell evasion by Murid Herpesvirus-4 (MuHV-4) results in CD8^+^ T cells controlling epithelial but not myeloid cell infection and gives CD4^+^ T cells an essential role in host defence [13]. CMVs are shed from the SG, where electron microscopy indicates that MCMV is produced mainly by acinar cells [14]. These lack MHC II, SG MHC II expression being limited to interdigitating myeloid cells [15]. However MCMV reaches the acinar cells via infected migratory dendritic cells [16]. Thus, dendritic cell recognition by CD4^+^ T cells could reduce acinar cell infection indirectly. Even if the dendritic cells were not killed, CD4^+^ T cell engagement might deliver anti-viral signals and arrest their migration, thereby reducing their capacity to spread infection.

Analysis of how MCMV might evade CD4^+^ T cells has focussed on cytokines: low MHC II expression on infected cells is attributed to an inhibition of IFNγ signalling [17] and an induction of IL-10 [18] reducing MHC II transcription. The viral genes responsible have not been identified. M27 reduces IFNγ signaling through STAT-2, but from day (d)7 of infection M27^-^ MCMV is no less attenuated in IFNγ receptor-deficient than in wild-type mice [19], so its main effect seems to be on type I IFN. We show that MCMV, like HCMV, degrades MHC II in infected cells, and that this requires M78, a multi-membrane spanning viral protein with homology to chemokine receptors but without canonical signalling motifs. M78^-^ MCMV shows reduced virus production from infected macrophages, and poorly colonizes the SG [20–22]. Myeloid cells infected by M78^-^ but not wild-type (WT) MCMV expressed MHC II *in vivo*, and CD4^+^ T cell loss significantly reversed the M78-dependent defect in SG colonization. Therefore CD4^+^ T cell evasion is an important M78 function that acts in myeloid cells and is necessary for MCMV to colonize its main site of long-term shedding.

## Results

### MCMV degrades MHC II in infected cells

**Fig 1:**
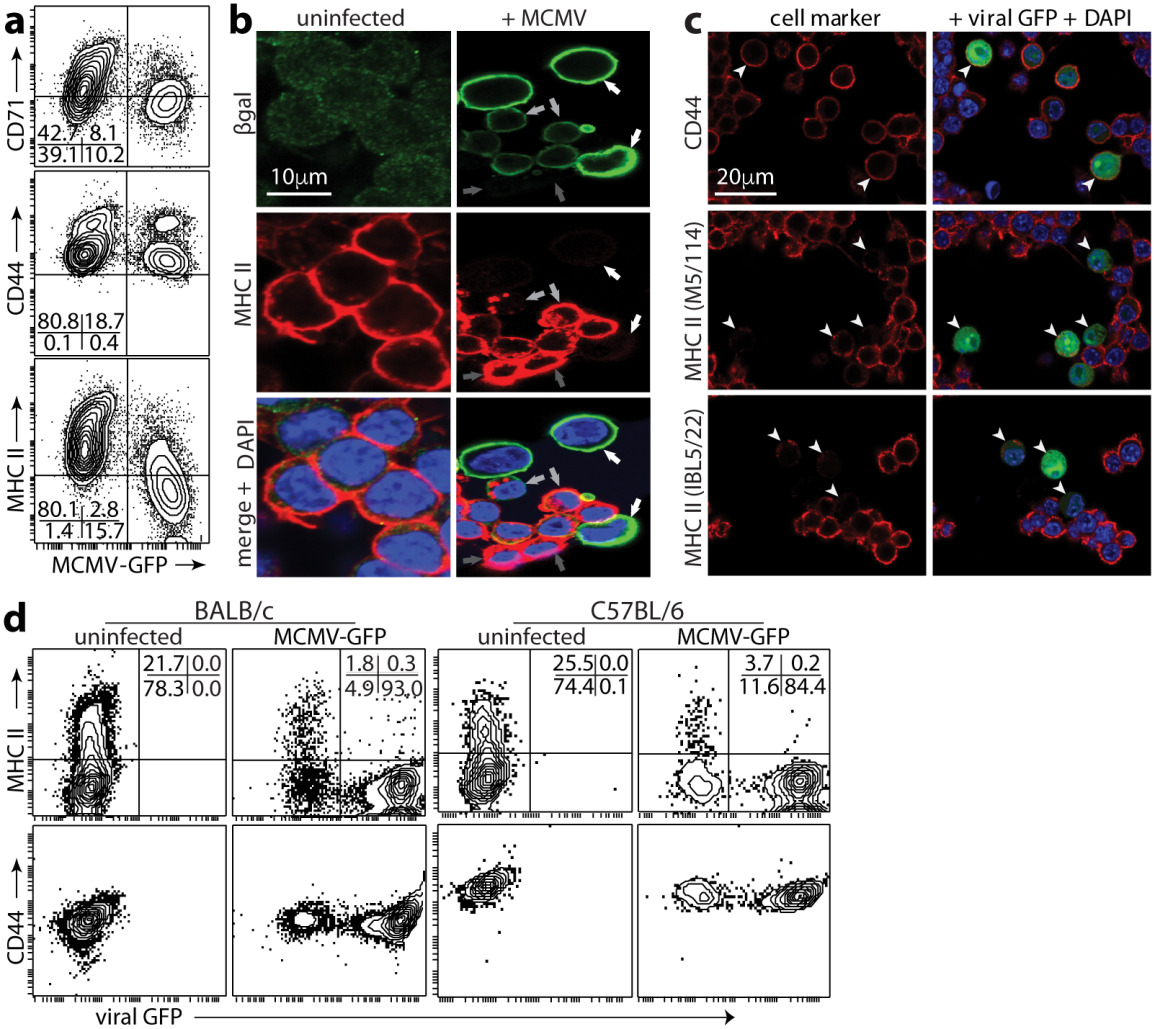
MCMV degrades MHC II. **a**. RAW-C2TA cells infected with MCMV-GFP (0.5 p.f.u. / cell, 48h) were analysed for surface MHC II by flow cytometry. Mean fluorescence intensity was >10-fold lower on GFP^+^ cells than on GFP^-^. CD44 and CD71 mean fluorescence intensity of GFP^+^ cells was reduced <2-fold. Numbers show % cells in each quadrant. Each data set represents at least 3 experiments. **b**. RAW-C2TA cells were uninfected or infected with MCMV-βgal (1 p.f.u. / cell, 72h), then fixed, permeabilized and stained for βgal and MHC II. White arrows show example MHC II^-^ infected cells; dark grey arrows show MHC II^+^ uninfected cells in the same cultures; light grey arrows show weakly βgal^+^ cells with MHC II in internal vesicles. Significantly fewer MCMV^+^ cells (<10%, n>200) than MCMV^-^ cells (>90%, n>200) were MHC II^+^ (p<10^-4^ by Fisher's exact test). >100 weakly βgal^+^ cells showed MHC II redistribution. **c**. RAW-C2TA cells were infected as in **b**. MHC II^+^ cells were identified with conformation-dependent (M5/114) and -independent (IBL5/22) antibodies. Arrows show example infected cells. Significantly fewer GFP^+^ (<15%, n>100) than GFP^-^ cells (>90%, n>100) were M5/114^+^ and IBL5/22^+^ (p<10^-4^ by Fisher's exact test). **d**. Peritoneal macrophages were recovered from BALB/c or C57BL/6 mice 72h after i.p. thiglycollate. Non-adherent cells were discarded. The remaining cells were infected or not with MCMV-GFP (1 p.f.u. / cell, based on total cell numbers before adherence). 48h later surface MHC II and CD44 were analysed by flow cytometry. Numbers show % total cells in each quadrant, collecting >5000 cells. In uninfected cultures, macrophages were 22% (BALB/c) and 26% (C57BL/6) MHC II^+^. In infected cultures, GFP^-^ macrophages were 27% (BALB/c) and 24% (C57BL/6) MHC II^+^, while GFP^+^ macrophages were 0.3% (BALB/c) and 0.2% (C57BL/6) MHC II^+^ (p<10^-4^ by X^2^ test).

Most myeloid cells express MHC II inducibly rather than constitutively. IFNγ induces MHC II expression but also inhibits MCMV replication [9, 23]. To track viral effects on MHC II without this complication, we induced MHC II in RAW-264 monocytes (normally MHC II^-^) by expressing the MHC II transactivator (C2TA), which acts down-stream of IFNγ [24]. RAW-C2TA cells were constitutively MHC II^+^. When they were exposed to MCMV-GFP, GFP^+^ cells lost MHC II but not CD44 or CD71 (Fig 1a). Immunostaining cells exposed to β-galactosidase (βgal)^+^ MCMV (Fig 1b) similarly showed normal or increased MHC II expression in βgal^-^ (uninfected) cells and MHC II loss in strongly βgal^+^ cells. In weakly βgal^+^ cells MHC II was clumped and internalized, suggesting that this was an intermediate stage in down-regulation.

Antibody IBL5/22, which recognizes a conformation-independent MHC II epitope, gave equivalent results to antibody M5/114 (Fig 1c), so MCMV caused MHC II degradation rather than just denaturation. MCMV also reduced MHC II on thioglycollate-induced peritoneal macrophages of BALB/c and C57BL/6 mice (Fig 1d). Thus the degradation did not depend on constitutive C2TA expression, and applied across at least 2 MHC II haplotypes.

### MCMV removes MHC II from the cell surface and degrades it in acidic endosomes

**Fig 2:**
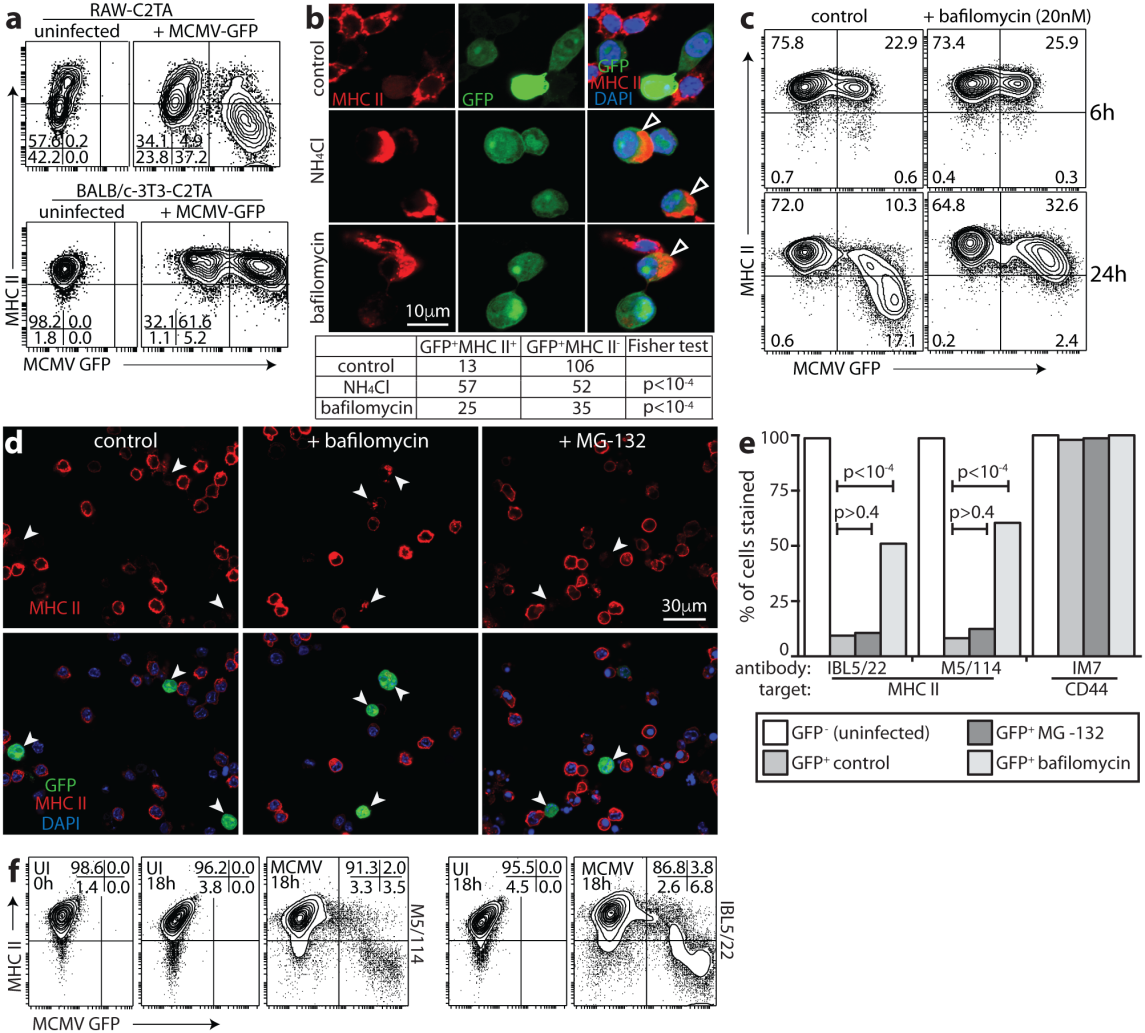
Viral MHC II degradation occurs in low pH endosomes. **a**. RAW-C2TA and BALB/c-3T3-C2TA cells were infected or not with MCMV-GFP (3 p.f.u. / cell and 0.5 p.f.u. / cell respectively, as MCMV more efficiently infects fibroblasts than RAW-264 cells). 18h later surface MHC II was assayed by flow cytometry. Unlike RAW-C2TA macrophages, infected 3T3-C2TA fibroblasts retained MHC II expression. **b**. RAW-C2TA cells infected or not with MCMV-GFP (1 p.f.u. / cell, 24h) were exposed (18h) to 100mM ammonium choride, 20nM bafilomycin or medium alone (control), then fixed, permeabilized and stained for MHC II. Viral GFP was visualized directly. Nuclei were stained with DAPI. Arrows show cells with rescued MHC II. The table quantitates MHC II expression for GFP^+^ cells. **c**. RAW-C2TA cells were infected with MCMV-GFP (3 p.f.u. / cell, 2h). Then bafilomycin (20nM) was added or not (control), and the cells cultured for a further 6h or 24h. They were then stained for MHC II with Alexa647-conjugated mAb M5/114 and analysed by flow cytometry. Virus-expressed GFP was assayed directly. Numbers show the % of total cells in each quadrant. **d**. RAW-C2TA cells were infected with MCMV-GFP (1 p.f.u. / cell, 72h), treated with bafilomycin (20nM) or the proteasome inhibitor MG-132 (50μM), and 18h later stained for MHC II. Arrows show example infected cells. The MHC II rescued by bafilomycin had an endosomal distribution. MG-132 gave no rescue. Representative images are shown. **e**. Total cell counts across at least 10 fields of view are shown for cells as illustrated in d (>200 cells per bar). Statistical comparison was by Fisher’s exact test. **f**. RAW-C2TA cells were incubated (1h, 37°C) with M5/114 or IBL5/22 rat mAbs to MHC II, washed to remove unbound antibody, infected (MCMV, 18h) or not (UI, 18h) with MCMV-GFP (2 p.f.u. / cell), cultured overnight (18h, 37°C), stained at 4°C with anti-rat IgG pAb and analysed by flow cytometry. Control untreated cells (UI, 0h) were stained in parallel for surface MHC II using M5/114 and anti-rat IgG pAb without overnight culture.

Unlike RAW-C2TA cells, MCMV-infected fibroblasts expressing C2TA preserved MHC II (Fig 2a). This result suggested that MHC II degradation might require macrophage-specific, acidic endosomes. To test the need for low pH, we exposed infected RAW-C2TA cells to ammonium chloride or bafilomycin, which inhibit endosomal acidification. Both treatments significantly rescued MHC II expression (Fig 2b and 2c). By contrast the proteasome inhibitor MG-132 had no effect (Fig 2d and 2e).

Complete MHC II loss from infected cells suggested that in addition to any effect on nascent protein, MCMV removed mature MHC II from the plasma membrane. To test this idea, we incubated RAW-C2TA cells with an MHC II-specific antibody (1h, 4°C), using excess antibody to minimize cross-linking and using rat antibodies to avoid binding by MCMV Fc receptors. We washed off unbound antibody, infected the cells or not overnight (18h, 37°C), then added a fluorescently labelled secondary antibody (1h, 4°C) and assayed its binding by flow cytometry (Fig 2f). Thus, we assayed MHC II that was on the plasma membrane before infection (for primary antibody binding) then retained there (for secondary antibody binding). We also stained MHC II on uninfected cells without a 37°C incubation (1h, 4°C) (t = 0h). The surface MHC II of uninfected RAW-C2TA cells was similar at t = 0h and t = 18h, indicating that its turnover is normally slow. By contrast MCMV-infected (GFP^+^) cells showed a marked loss of surface-tagged MHC II over 18h. Therefore MCMV removed MHC II from the plasma membrane of infected cells. As RAW-C2TA cells maintained surface MHC II for at least 18h, reduced synthesis—for example through transcriptional suppression—could not explain the down-regulation. Minimal MHC II down-regulation by MCMV in BALB/c-3T3-C2TA cells (Fig 2a) and rescue by bafilomycin (Fig 2b–2e) also argued against transcriptional suppression making a significant contribution.

### MHC II degradation by MCMV requires M78

**Fig 3:**
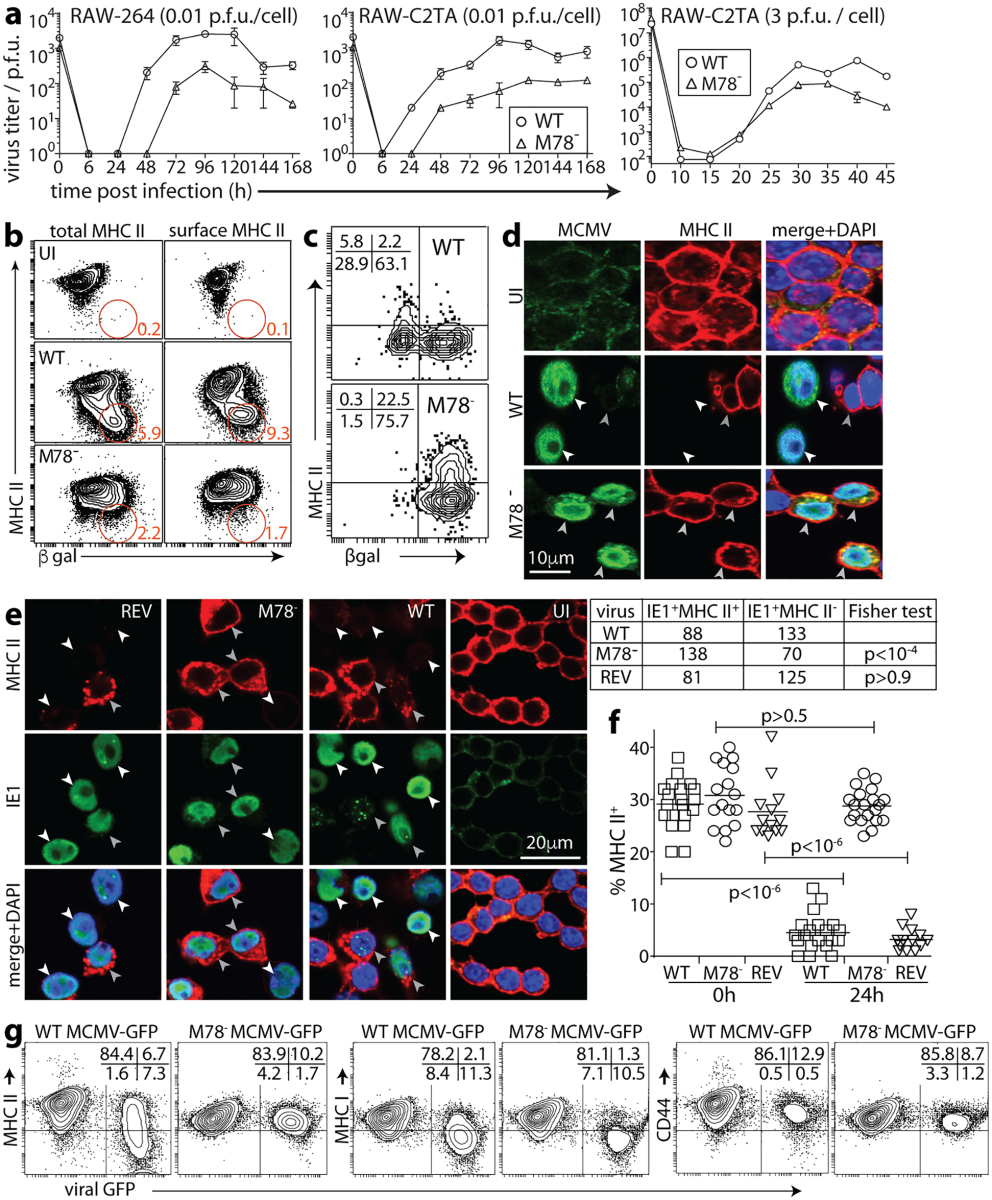
MHC II degradation by MCMV requires M78. **a**. RAW-264 and RAW-C2TA cells were infected at low multiplicity (0.01 p.f.u. / cell, 1h) and washed x3 in PBS. RAW-C2TA cells were also infected at high multiplicity (3 p.f.u. / cell) by centrifuging virus onto the cells (400 x *g*, 30min), then washed x 3 in PBS. The cells were cultured in complete medium, and at each time point, triplicate cultures were plaque-assayed for infectious virus. Symbols show mean ± SEM. Time = 0 shows the calculated virus input. M78^-^ MCMV had similar growth defects in MHC II^+^ and MHC II^-^ cells. **b**. RAW-C2TA cells were infected with WT or M78^-^ βgal^+^ MCMV (1 p.f.u. / cell, 72h), or left uninfected (UI), then stained for surface MHC II (non-permeabilized) before fixation, permeabilization and staining for βgal; or fixed, permeabilized then stained for MHC class II plus βgal (permeabilized). MHC II either just at the cell surface (non-permeabilized) or also in the cell (permeabilized) was assayed by flow cytometry. Numbers show % total cells in each gated region. This was significantly less for M78^-^ than for WT MCMV (p<10^-4^ by X^2^ test.) Total βgal^+^ cell numbers were comparable. **c**. Peritoneal macrophages from thioglycollate-treated BALB/c mice were infected with WT or M78^-^ βgal^+^ MCMV (1 p.f.u. / cell, 48h), then fixed, permeabilized, stained with antibodies to MHC II and βgal, and analysed by flow cytometry. Numbers show % total cells in each quadrant. Significantly more GFP^+^ cells were MHC II^+^ with M78^-^ than with WT MCMV (p<10^-4^ by X^2^ test). **d**. RAW-C2TA cells infected as in **b** were fixed, permeabilized, stained for MHC II and for MCMV antigens with polyclonal immune serum, then imaged by confocal microscopy. For WT MCMV, white arrows show infected cells and the grey arrow an uninfected cell. For M78^-^ MCMV, arrows show infected cells. M78^-^ but not WT infected cells showed MHC II^+^MCMV^+^ vesicles just below the plasma membrane. Significantly fewer MCMV^+^ cells were MHC II^+^ for WT (4/59 cells) than for M78^-^ infection (62/69 cells) (p<10^-4^ by Fisher's exact test). **e**. RAW-C2TA cells were infected with WT, M78^-^ or revertant (REV) viruses (1 p.f.u. / cell, 72h), or left uninfected (UI). They were then fixed, permeabilized and stained for MHC II plus MCMV IE1. White arrows show infected cells that have lost MHC II expression; grey arrows show cells that retain it. Where WT and REV-infected cells retained some MHC II, corresponding presumably to an earlier stage of infection, it showed an altered, endosomal distribution. The table gives total counts for >30 fields of view per sample. **f**. Macrophages grown from bone marrow cells were infected with WT, REV or M78^-^ MCMV (2 p.f.u. / cell, 24h), then fixed and stained for MHC II. Symbols show counts for individual fields of view. Bars show means. WT and REV infections caused significant MHC II^+^ cell loss; M78^-^ infection did not. **g**. RAW-C2TA cells were infected with WT or M78^-^ (deletion mutant) MCMV-GFP (1 p.f.u. / cell, 72h), then analysed by flow cytometry for surface glycoproteins. % total cells in each quadrant is shown. M78 disruption significantly reduced loss of MHC II (p<10^-4^ by X^2^ test) and not MHC I. CD44 was maintained.

MHC II loss from the cell surface, and the intracellular distribution of the MHC II rescued by ammonium chloride or bafilomycin (Fig 2), argued that MCMV initiates MHC II degradation by relocalizing it to endosomes. The MCMV M78 is highly endocytic [21], so we tested its contribution, infecting RAW-C2TA cells with WT or M78^-^ MCMV (Fig 3). M78^-^ MCMV is reported to grow normally in fibroblasts and poorly in macrophages [20, 22]. We found impaired infectious virus production in RAW-264 cells after low multiplicity infection, with less defect after higher multiplicity infection (Fig 3a). There was no difference between MHC II^+^ and MHC II^-^ RAW-264 cells. There was no obvious defect in RAW-264 cell infection as measured by viral β-gal expression (Fig 3b), or by flow cytometry of viral GFP expression (S1a Fig), and RAW-C2TA cells infected by WT or M78- MCMV showed no obvious difference in viral or MHC II transcription (S1b and S1c Fig).

Flow cytometry of WT MCMV-infected cells showed a marked loss of both surface (non-permeabilized), and total (permeabilized) MHC II (Fig 3b). M78^-^ MCMV infection little affected either. MHC II loss from peritoneal macrophages also required M78 (Fig 3c). Identifying infected cells by immunofluorescent staining with a polyclonal anti-MCMV antibody (Fig 3d) or with an IE1-specific antibody (Fig 3e) confirmed that MHC II loss was M78-dependent. We also tested bone marrow-derived macrophages (Fig 3f). When uninfected, approximately 30% of these were MHC II^+^; when infected with WT or M78^+^ REV MCMV, expression decreased to <5%; and when infected with M78^-^ MCMV there was no decrease.

Flow cytometry of cells infected with an independent M78^-^ mutant (Fig 3g) confirmed that MHC II loss was M78-dependent. MHC I loss was contrastingly M78-independent. In H2^d^ cells this depends primarily on M06, which degrades MHC I in lysosomes [25]. Therefore M78 was not required for all MCMV-driven, endosomal protein degradation. As M78^+^ MCMV did not down-regulate CD71 (Fig 1a), which cycles through early endosomes, or CD44, M78 did not generally promote glycoprotein endocytosis and loss.

### M78 relocalizes MHC II to endosomes

**Fig 4:**
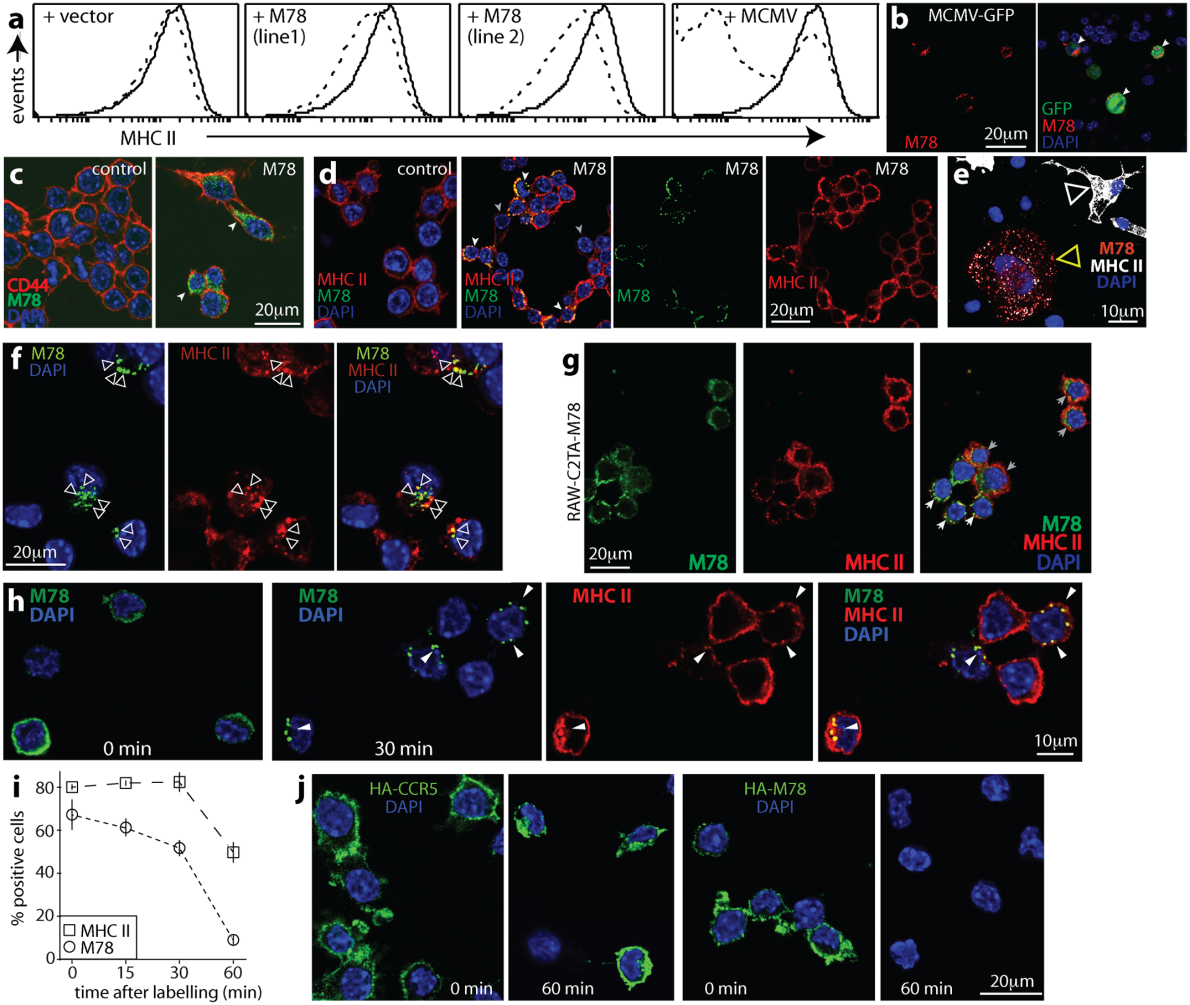
M78 relocalizes MHC II. **a**. Cloned RAW-C2TA cells were transduced with vector alone or with M78 (line 1 and line 2), or infected with MCMV-GFP (+ MCMV, 1 p.f.u. / cell, 72h), then stained for MHC II (dashed lines). Solid lines show uninfected, untransduced RAW-C2TA cells. The biphasic population with MCMV corresponds to GFP^+^ (MHC II^-^) and GFP^-^ (MHC II^+^) cells. M78 transfection had much less effect than MCMV infection on cell surface MHC II expression. **b**. MCMV-GFP-infected RAW-C2TA cells were stained for M78. GFP was visualized directly. Nuclei were stained with DAPI. Arrows show example infected cells with M78 in vesicles. **c**. Untransduced (control) and M78-transduced RAW-C2TA cells were stained for M78 and CD44. Arrows show M78 staining, which did not co-localize with CD44. **d**. M78-transduced RAW-C2TA cells were stained for M78 and MHC II. M78 occupied intracellular vesicles, as did MHC class II in M78^+^ cells. White arrows show examples of co-localization. Grey arrows show M78^-^ cells with MHC II on the plasma membrane. **e**. Macrophages grown from bone marrow were infected with WT MCMV (1 p.f.u. / cell, 24h), then fixed and stained for M78 and MHC II. The white arrow shows a typical uninfected MHC II^+^ cell. The yellow arrow shows a typical infected cell with MHC II in vesicles. >80% of vesicles were M78^+^MHC II^+^, <20% were M78^+^MHC II^-^ and <1% were M78^-^MHC II^+^ (n>300). Thus there was significant endosomal M78 / MHC II co-localization (p<10^-4^ by Fisher's exact test). **f**. RAW-C2TA cells were transfected with a plasmid expressing HA-tagged M78. 3d later they were fixed, permeabilized and stained for M78 and MHC II. Nuclei were stained with DAPI. Arrows show examples of M78 and MHC II co-localizing in internal vesicles. **g**. Cloned RAW-C2TA-M78 cells were stained for MHC II and M78. White arrows show M78^+^ cells with MHC II in vesicles; grey arrows show cells with diffuse M78 staining and MHC II not relocalized. M78/MHC II co-localization was observed in >30% of transduced cells (n>500) and not in untransduced controls. **h**. RAW-C2TA cells were infected with HA-M78^+^ MCMV (4h, 37°C), incubated with rabbit anti-HA and rat anti-MHC II IgG (1h, 4°C), then washed and incubated at 37°C for the times indicated before fixation, permeabilization and staining with anti-rat (red) and anti-rabbit (green) antibodies. Nuclei were stained with DAPI. Stainings show the change in distribution of surface-labelled M78 from 0 to 30min, and at 30min M78 co-localization with internalized MHC II (yellow, arrows). **i**. Summary of staining results from **h** shows similar kinetics of surface-labelled M78 and MHC II loss. Cells were scored as stained or not regardless of internalization. Each point shows mean ± SEM of 5 fields of view and >100 cells counted. **j**. RAW-C2TA cells were transfected with plasmid expressing HA-tagged M78, or HA-tagged CCR5 as a control. 3d later they were incubated with rabbit anti-HA IgG (1h, 4°C), then washed and either fixed and permeabilized at once (0 min) or first incubated at 37°C (60 min). All cells were then stained with for rabbit IgG (green). Nuclei were stained with DAPI. HA-CCR5 detection was maintained over 60min, whereas HA-M78 detection was lost.

RAW-C2TA cells expressing M78 via retroviral transduction showed some MHC II loss but much less than that of infected cells (Fig 4a). Both MCMV-infected RAW-C2TA cells (Fig 4b) and RAW-C2TA cells transduced with an M78^+^ retrovirus (Fig 4c and 4d) expressed M78 in internal vesicles—previous studies have identified it in early endosomes [21]. In transduced cells, CD44 remained on the plasma membrane whereas MHC II relocalized to M78^+^ vesicles. MCMV-infected bone marrow-derived macrophages (Fig 4e) and RAW-C2TA cells transiently transfected with an M78 expression plasmid (Fig 4f) similarly showed MHC II / M78 co-localization in vesicles.

Surprisingly, despite M78-transduced cells being readily obtained and despite retroviral transduction generally giving uniformly strong recombinant gene expression [26], M78-transduced RAW-C2TA cells showed variable M78 expression (Fig 4d), as did RAW-C2TA-M78 cell clones and an independently-derived RAW-C2TA-M78 line (Fig 4g). Cells with strong M78 expression redistributed MHC II, but puromycin-resistant cells that expressed less M78 had less effect on MHC II. To understand why, we tracked M78 from the cell surface. We infected RAW-C2TA cells with MCMV expressing HA-tagged M78 (4h, 37°C), labelled them with HA-specific rabbit IgG and MHC II-specific rat IgG (1h, 4°C), washed off excess antibody, then incubated the cells at 37°C for different times before fixation, permeabilization and staining with anti-rabbit IgG and anti-rat IgG labelled secondary antibodies. M78 tagged by antibody at the infected cell surface was rapidly endocytosed (Fig 4h). It co-localized with MHC II in internal vesicles, then became undetectable, as did surface-tagged MHC II (Fig 4i). Transfected HA-M78 was also lost rapidly, whereas transfected HA-CCR5 was preserved (Fig 4j). Therefore rapid M78 and MHC II endocytosis appeared to be accompanied by M78 degradation, and in infected cells also by MHC II degradation. In transduced cells, even high level M78 expression did not lead to MHC II loss (Fig 4d).

### CD4^+^ T cell loss significantly rescues *in vivo* infection by M78^-^ MCMV

**Fig 5:**
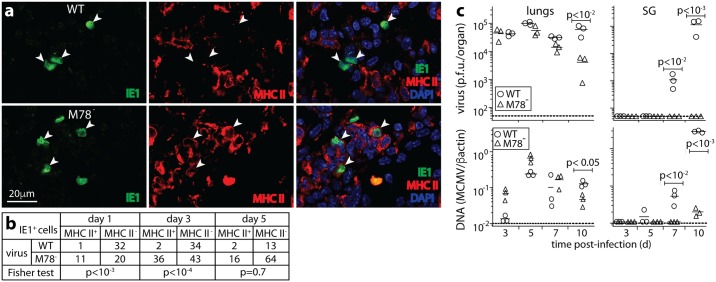
M78^-^ MCMV replication *in vivo*. **a**. BALB/c mice were given i.n. WT or M78^-^ MCMV (3x10^4^ p.f.u.). 3d later lung sections were stained for MCMV IE1 and MHC II. Nuclei were stained with DAPI. Arrows show infected cells. **b**. Quantitation of staining as in **a**, for sections from 3 mice. Few WT infected cells were MHC II^+^. At d1 and d3, significantly more M78^-^-infected cells were MHC II^+^. **c**. Mice were infected as in **a**. Lungs and salivary glands were plaque-assayed for infectious virus, and QPCR-assayed for viral genomes relative to cellular (βactin) genomes. Bars show means, other symbols show individual mice. Dashed lines show assay sensitivity limits. Significant differences are indicated.

As M78 was necessary for MCMV-driven degradation, M78^-^ MCMV provided an opportunity to understand what CD4^+^ T cell evasion contributes to host colonization *in vivo*. We gave mice intranasal (i.n.) WT or M78^-^ MCMV (Fig 5). In the lungs MCMV infects myeloid cells and type II alveolar epithelial cells [27]. Both can express MHC II [13]. After 1-3d few WT-infected lung cells expressed MHC II, whereas many of those infected by M78^-^ MCMV did so (Fig 5a and 5b). Therefore M78 also down-regulated MHC II *in vivo*.

M78 has been studied as an MCMV gene of unknown function: M78^-^ MCMV given intraperitoneally (i.p.) shows reduced liver, spleen and SG infections [20]; given i.n. it shows reduced lung and SG infections [22]. Rat CMV lacking its M78 homolog (R78) is also attenuated *in vivo* [28]. Plaque assays of infectious virus and QPCR of viral DNA showed normal acute lung infection. This reflected presumably that myeloid cells are not a major source of acute virus production in the lungs [27]. However M78^-^ MCMV was cleared faster from the lungs, and showed a marked defect in SG infection (Fig 5c).

**Fig 6:**
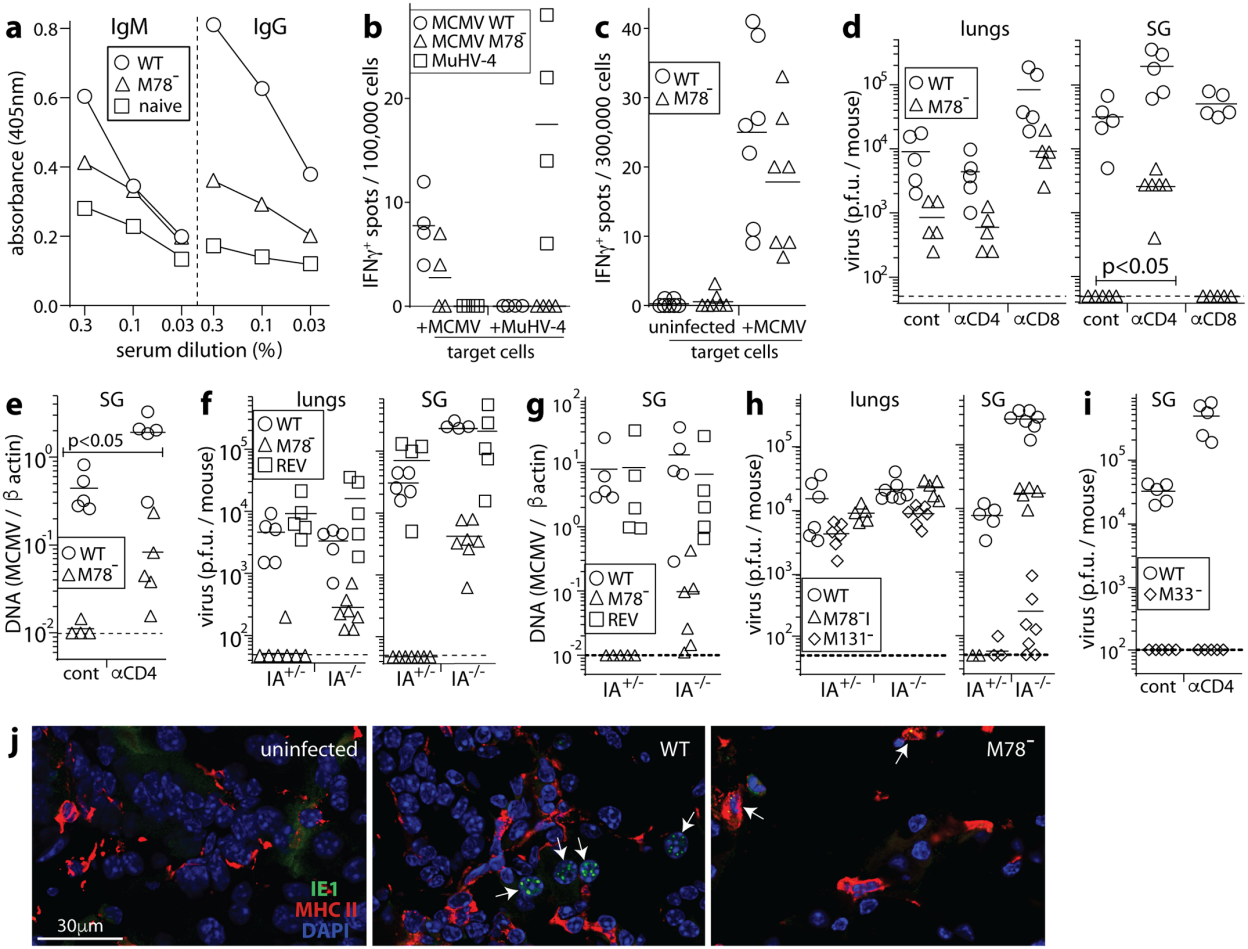
Significant M78^-^ MCMV rescue by CD4^+^ T cell loss. **a**. C57BL/6 mice were given WT or M78^-^ MCMV i.n. (3x10^4^ p.f.u.). 56d later sera were assayed for MCMV-specific IgG and IgM by ELISA. Naive = age-matched, uninfected controls. Each point shows the mean of results for 7 mice. M78^-^ MCMV elicited significantly less IgG response than WT (p<0.01). **b**. C57BL/6 mice were given WT or M78^-^ MCMV, or as a control MuHV-4 i.n. (3x10^4^ p.f.u.). 56d after MCMV infection or 10d after MuHV-4 infection, CD4^+^ T cells were purified from splenocytes, pooled from 2 mice per group, by depleting other cells with magnetic beads (Untouched mouse CD4 cell kit, Thermofisher). IFNγ production in response to MCMV-exposed or MuHV-4-exposed naive syngeneic spleen cells (1 p.f.u. / cell) was measured by ELIspot assay. Symbols show replicate wells, bars show means. **c**. C57BL/6 mice were given WT or M78^-^ MCMV i.n. (3x10^4^ p.f.u.). 56d later IFNγ production by splenocytes exposed to uninfected or MCMV-exposed naive syngeneic spleen cells was measured by ELIspot assay. Symbols show individual mice, bars show means. CD4^+^ T cell responses to WT and M78^-^ MCMV were not significantly different. **d**. BALB/c mice were depleted of CD4^+^ or CD8^+^ T cells (αCD4, αCD8) or left undepleted (cont), then given i.n. WT or M78^-^ MCMV (3x10^4^ p.f.u.). 10d later lungs and SG were plaque assayed for infectious virus. Symbols show individuals, bars show means. In lungs, immune depletions did not significantly change the ratio of WT to M78^-^ titers. In SG, CD4^+^ T cell depletion significantly reduced this ratio. **e**. Viral DNA loads of SG in **d** were determined by QPCR. Again CD4^+^ T cell depletion significantly reduced the ratio of WT to M78^-^ infection, that is significantly reversed the M78^-^ infection defect. **f**. Immunocompetent (IA^+/-^) and MHC II^-^ (IA^-/-^) C57BL/6 mice were given WT, M78^-^ or revertant (REV) MCMV i.n. (3x10^4^ p.f.u.). At d10 lungs and SG were plaque-assayed for infectious virus. Symbols show individual mice, bars show means. For both lungs (p<0.05) and SG (p<0.01), CD4^+^ T cell loss (IA^-/-^ mice) significantly increased M78^-^ plaque titers relative to WT or REV. **g**. For the mice in **f**, CD4^+^ T cell loss significantly increased M78^-^ viral DNA loads in SG relative to WT or REV (p<0.05). **h**. IA^-/-^ and IA^+/-^ mice were given i.n. WT, M131^-^ or M78 deletion mutant (M78^-^I) MCMV. At d10 lungs and SG were plaque assayed for infectious virus. Symbols show individuals, bars show means. The ratio of M78^-^I/WT salivary gland titers was significantly higher in IA^-/-^ than IA^+/-^ mice (p<0.01), while the ratio of M131^-^/WT salivary gland titers was reduced. **i**. BALB/c mice were depleted of CD4^+^ T cells as in **d**, then given WT or M33^-^ MCMV i.n.. After 10d SG were plaque assayed for infectious virus. Symbols show individuals, bars show means. CD4^+^ T cell depletion increased WT MCMV SG infection but failed to rescue SG infection by M33^-^ MCMV. **j**. BALB/c mice were depleted of CD4^+^ T cells and given WT or M78^-^ MCMV i.n. as in **d**. At d10 salivary gland sections were stained for MHC II and MCMV IE1. UI = uninfected control. Arrows show example infected cells (speckled nuclear IE1 staining). These cells were all MHC II^-^ with WT MCMV and MHC II^+^ with M78^-^ MCMV. Images are representative of 6 mice per group.

Antibody responses to M78^-^ MCMV were significantly lower than those to WT infection (Fig 6a), consistent with M78^-^ viral loads being lower. ELIspot assays (Fig 6b and 6c) showed no obvious difference in CD4^+^ T cell response between M78^-^ and WT MCMV. We assessed the functional contribution of CD4^+^ T cells to M78^-^ MCMV attenuation by infecting BALB/c mice depleted of T cell subsets (Fig 6d). CD8^+^ T cell depletion increased M78^-^ MCMV titers in the lungs at d10. However it increased WT titers by a similar amount (p>0.5). It did not significantly affect SG infection. Therefore M78^-^ MCMV attenuation was not due to better control by CD8^+^ T cells.

CD4^+^ T cell depletion did not alter lung infection. However it increased SG infection by M78^-^ MCMV. Some M78-dependent defect remained relative to WT, but unlike with CD8^+^ T cell depletion there was a significant relative increase in M78^-^ virus titers (p<0.04). CD4^+^ T cell depletion also increased M78^-^ viral genome loads relative to WT (Fig 6e). Therefore CD4^+^ T cell evasion by M78 was important for salivary gland colonization by MCMV.

To confirm this result, we infected MHC II-deficient C57BL/6 mice (IA^-/-^) with WT, M78^-^ mutant or M78^+^ revertant (REV) viruses (Fig 6f and 6g). At d10, virus titers in the lungs and SG of immunocompetent mice (IA^+/-^) were significantly lower for M78^-^ MCMV than for WT or REV. IA^-/-^ lungs showed higher M78^-^ plaque titers, and the increase in M78^-^ titer with CD4^+^ T cell loss (IA^-/-^ mice) was significantly greater than for WT or REV MCMV (p<0.05). CD4^+^ T cell loss also increased M78^-^ titers in SG, relative to both WT and REV (p<0.004), as well as M78^-^ viral DNA loads (p<0.01). To exclude a generally greater effect of CD4^+^ T cell depletion on low level SG colonization, we compared our untagged M78 deletion mutant (M78^-^I) with M131^-^ MCMV [29], which also poorly colonizes SG (Fig 6h). Again M78^-^ titers in SG showed a greater defect relative to WT in IA^+/-^ than in IA^-/-^ mice (p<0.01), indicating significant rescue by CD4^+^ T cell loss. By contrast M131^-^ titers in SG showed a greater defect relative to WT in IA^-/-^ mice. Nor did CD4^+^ T cell depletion rescue SG infection by i.n. M33^-^ MCMV (Fig 6i). Therefore CD4^+^ T cell loss specifically rescued SG infection by M78^-^ MCMV.

### SG cells infected by M78^-^ but not WT MCMV are MHC II^+^

Salivary acinar cells are MHC II^-^ [14] (Fig 6j, uninfected). However dendritic cells disseminate i.n. MCMV, appear in the SG before acinar cells become infected [16], and can express MHC II. For at least 2 weeks after i.n. MCMV >80% of infected cells in the SG are CD11c^+^ (myeloid) rather than Aquaporin V^+^ (acinar). At d10 after WT infection of CD4^+^ T cell-depleted mice, all infected cells (n>100, counting samples from 6 mice) were MHC II^-^ (Fig 6j, WT). By contrast those infected by M78^-^ MCMV were MHC II^+^ (Fig 6j, M78^-^). Without CD4^+^ T cell depletion, no M78^-^ infected cells were seen. Therefore M78 promoted SG colonization before acinar cell infection, by guarding infected, disseminating myeloid cells against CD4^+^ T cell engagement.

## Discussion

M33 of MCMV [30], and UL33 and US28 of HCMV [31] encode chemokine receptor homologs that signal constitutively. M78 and its equivalents in HCMV (UL78) and rat CMV (R78) have not been shown to signal, nor to bind chemokines, implying that they have other functions. M78 relocalized MHC II to endosomes and was required for MCMV-driven MHC II degradation. M78^-^ MCMV also produced less infectious virus than did WT MCMV from MHC II^+^ and MHC II^-^ RAW-264 cells, so MHC II degradation is unlikely to be the only M78 function. However CD4^+^ T cell loss significantly rescued poor SG infection by M78^-^ MCMV. Therefore M78-dependent CD4^+^ T cell evasion made a demonstrably important contribution to host colonization.

MCMV reaches the SG via infected, migratory dendritic cells [16, 32, 33]. With WT MCMV, these cells lacked detectable MHC II. With M78^-^ MCMV, no infected cells were seen in SG of immunocompetent mice, and CD4^+^ T cell loss led to the appearance of MHC II^+^ infected cells. Thus M78 promoted SG infection by increasing virus transport to the SG, presumably protecting infected dendritic cells against CD4^+^ T cell engagement. Impaired virus production by M78^-^ infected myeloid cells might also impair infection transfer to SG acinar cells, but the primary defect was in the initial arrival of infected cells. Infected dendritic cells drive the systemic spread of i.n. MCMV [16], but type 2 alveolar epithelial cells appear to produce most of the infectious virus in lungs [27]. Thus, normal acute lung infection by M78^-^ MCMV was consistent with a myeloid cell-focussed defect. M78 could be considered a systemic spread-specific infection module that couples CD4^+^ T cell evasion to virus production in myeloid cells.

Despite MHC II degradation by CMVs, CD4^+^ T cells play an important role in infection control [7, 8]. We hypothesize that protective CD4^+^ T cells normally respond to antigen on uninfected presenting cells and act indirectly. This idea is supported by salivary acinar cells lacking MHC II [14], by most MHC II presenting cell-exogenous rather than cell-endogenous antigens, and by MCMV disrupting other presenting functions in infected myeloid cells [34, 35]. The failure of M78 disruption to increase MCMV-specific CD4^+^ T cell responses was consistent with uninfected presenting cells priming most CD4^+^ T cells. It follows that protective CD4^+^ T cells may not directly recognize CMV-infected cells, but rather recruit and activate other anti-viral effectors with independent modes of recognition, for example NK cells.

What then does MHC II degradation in infected cells achieve? Because MCMV exploits normal dendritic cell migration to spread [16], infected cells are likely to encounter CD4^+^ T cells in lymph nodes. CD4^+^ T cell recognition of MHC II plus antigen on infected dendritic cells would not necessarily lead to killing, as CD4^+^ T cells primarily activate rather than kill engaged presenting cells. (CD8^+^ T cell evasion is probably more important for infected cell survival.) However CD4^+^ T cell-derived cytokines can have anti-viral effects [9], and CD4^+^ T cell engagement would reduce the migration of infected myeloid cells and so their capacity to spread infection. Increased CD4^+^ T cell engagement would also promote local antibody responses and innate effector recruitment. Thus MHC II expression on infected myeloid cells, even if presenting non-viral antigens, would open up a new front of host defence, with more precise targeting of recruited effectors to infected cells. By removing MHC II from infected myeloid cells, M78 isolated them from any CD4^+^ T cell engagement, promoting systemic infection spread and making MCMV-infected cells harder for CD4^+^ T cell-dependent defences to find.

## Materials and methods

### Mice

BALB/c, C57BL/6 and IA^-/-^ mice [36] were maintained at University of Queensland animal units, and infected i.n. at 6–12 weeks old (3x10^4^ p.f.u. in 30μl, under anesthesia). IA^-/-^ mice for breeding were kindly provided by Prof. Geoff Hill (Queensland Institute for Medical Research). We depleted CD4^+^ / CD8^+^ T cells with mAbs GK1.5 / 2.43 (Bio X Cell, 200μg/mouse/48h, from 96h pre-infection). Flow cytometry of spleen cells showed that the depletions were >95% complete (S2 Fig). Embryonic fibroblasts were obtained from 15–17d old embryos harvested from pregnant out-bred CD1 mice.

### Ethics statement

Experiments were approved by the University of Queensland Animal Ethics Committee (projects 301/13, 391/15 and 479/15) in accordance with the Australian code for the care and use of animals for scientific purposes, from the Australian National Health and Medical Research Council.

### Plasmids

M78 was amplified from K181 MCMV with Q5 polymerase (New England Biolabs), adding *Mfe*I and *Sal*I sites to its 5' and 3' ends, ligated into pMSCV-IRES-PURO [37] and verified by DNA sequencing. Expression plasmids for HA epitope-tagged-M78 and CCR5 are described [21]. Each HA tag was N-terminal and so extracellular when the protein spanned the plasma membrane.

### Cells

Peritoneal macrophages were obtained by peritoneal lavage 48h after i.p. 3% Brewer's thioglycollate. B cells were removed by adherence to plastic and washing with PBS. Recovered cells were >90% F4/80^+^CD19^-^. Macrophages were grown from bone marrow by culture with M-CSF-1 (10ng/ml, Peprotech). These cells, mouse embryo-derived fibroblasts, NIH-3T3 (American Type Culture Collection (ATCC) CRL-1658), 293T (ATCC CRL-3216), RAW-264 cells (ATCC TIB-71), RAW-264 cells transduced with the human MHC II transactivator to induce MHC II (RAW-C2TA) [38], BALB/c-3T3 and BALB/c-3T3 cells transduced with C2TA, were grown in Dulbecco’s modified Eagle’s medium with 2mM glutamine, 100IU/ml penicillin, 100μg/ml streptomycin and 10% fetal calf serum (complete medium). Retroviral transduction was by transfecting 293T cells with pMSCV-M78-PURO and a packaging plasmid [37], adding supernatants to cells with hexadimethrine bromide (10μg/ml), then selecting with puromycin (10μg/ml). RAW-C2TA cells were transfected by electroporation.

### Viruses

We used MCMV strain K181. Variants expressing GFP from the M131 intron (MCMV-GFP) [27] or βgal from the M33 intron (MCMV-βgal) [39]; with a premature stop codon in M131 (M131^-^) [29]; with a βgal cassette replacing M33 (M33^-^) [39]; with a βgal expression cassette at genomic coordinate 111681 (Genbank GU305914) disrupting M78 (M78^-^); and a revertant with an N-terminal HA epitope tag on M78 (REV) [21] are described. An independent M78 mutant (M78^-^I) was made by homologous recombination, deleting the ORF (coordinates 111084–112499). This mutation was also recombined into MCMV-GFP. Viruses were grown in NIH-3T3 cells. Viruses were plaque assayed on embryonic fibroblasts [27]. Statistical comparison was by Student's 2-tailed unpaired t test unless otherwise stated.

### Immunostaining

Organs were fixed in 1% formaldehyde-10mM sodium periodate-75mM L-lysine (24h, 4°C), equilibrated in 30% sucrose (18h 4°C), then frozen. 6μM sections were blocked with 0.3% Triton X-100 / 5% normal goat serum, then incubated (18h, 4°C) with mAbs to MHC II (rat, M5/114) and MCMV IE1 (mouse IgG_1_, CROMA101) [40]. Sections were washed x3 in PBS, incubated (1h, 23°C) with Alexa 488-goat anti-mouse IgG_1_ and Alexa 568-goat anti-rat IgG pAb plus DAPI (1μg/ml), then washed x3 in PBS, and mounted in ProLong Gold (Life Technologies). Cultured cells were adhered to coverslips, then fixed (2% formaldehyde, 30min), blocked in PBS / 0.1% Triton-X100 / 1% bovine serum albumin, then stained with antibodies to MHC II (M5/114 or IBL5/22), CD44 (rat mAb IM7), MCMV IE1 (CROMA101), βgal (chicken pAb, AbCam), MCMV (rabbit pAb), and M78 (rabbit pAb) [21]. Cells were washed x3 in PBS / 0.1% Tween-20, incubated with combinations of Alexa488-goat anti-chicken IgG pAb, Alexa488-goat anti-mouse IgG_1_, Alexa488- or Alex568-goat anti-rabbit pAb and Alexa568-goat anti-rat IgG pAb, plus DAPI (1μg/ml), then washed x3 in PBS / 0.01% Tween-20, x2 in PBS and mounted ProLong Gold. GFP fluorescence was visualized directly. Images were acquired with a Zeiss LSM510 microscope and analyzed with ImageJ.

### Flow cytometry

Cells were detached from plates, blocked with 1% BSA / 1μg/ml anti-CD16/32 (2.4G2), incubated (1h, 4°C) with antibodies to MHC II (APC-mAb M5/114), CD44 (biotin-IM7), MHC class I (biotin-34-5-8S), CD71 (biotin-C2, BD Biosciences), washed x2 in PBS, then incubated with fluorescein-streptavidin, washed x2 in PBS and analysed on an Accuri flow cytometer. GFP fluorescence was measured directly. To detect βgal, cells were fixed in 2% PFA after surface staining, then permeabilized in 70% ethanol, washed x2 and incubated with chicken anti-βgal followed by Alexa488-goat anti-chicken IgG pAb (AbCam). To test CD4^+^ and CD8^+^ T cell depletions, spleen cells were stained with antibodies to CD4 (fluorescein-RM4-4), and CD8β (phycoerythrin-H35-17.2) (BD Biosciences).

### Quantitative PCR

DNA was extracted from organs or blood (Wizard Genomic DNA Purification, Promega). MCMV coordinates 111218–111461 were amplified (LightCycler 480 SYBR green, Roche Diagnostics) and converted to genome copies by comparison with plasmid DNA amplified in parallel. Cellular DNA was quantified in the same samples by amplification of a β-actin gene segment. Viral DNA loads were normalized by cellular DNA loads.

### RT-PCR

RNA was extracted from cells (Ambion) and reverse-transcribed with an oligo-dT primer (New England Biolabs). MHC II, β2M cellular cDNAs and IE1 and M33 viral cDNAs were then amplified by PCR. To distinguish cDNA from genomic DNA, each primer pair spanned an intron. The primers were (5' to 3'): MHC II—GATGCCGCTCAACATCTTGCTC and CATCCACACAGCTTATTAGGAATG; β_2_M—TAGACCAAAGATGAGTAACTGCATC and GAGACTGATACATACGCCTGCAG; IE1—AACCGTCCGCTGTGACCTGAC and CGATGCGCTCGAAGATATCATTG; M33—TCAGGATGATCACCGTGTTGATG and GAAACTTCTTAACCTTTCCAACGG. PCR products were separated by electrophoresis on 2% agarose gels and visualized by staining with ethidium bromide.

## Supporting information

S1 Fig Normal M78^-^ MCMV infection of RAW-C2TA cells. **a**. BHK-21, NIH-3T3 cells and RAW-C2TA cells were infected with GFP^+^ WT or GFP^+^ M78^-^ MCMV at different multiplicities. GFP expression was then quantified by flow cytometry, after 18h for BHK-21 and NIH-3T3 cells, and after 24, 48 and 72 hours for RAW-C2TA cells. No significant difference in infection was observed between WT and M78^-^ MCMV.
**b**. RAW-C2TA cells were infected with WT or M78^-^ MCMV (3 p.f.u. / cell, 72h). RNA was then harvested, reverse transcribed with an oligo-dT primer and used to amplify MHC II, β_2_-microglobulin (β_2_M), the MCMV IE1 or the MCMV M33. Each primer set spanned an intron. The filled arrow shows the predicted size of the product amplified from spliced cDNA, and the open arrow that amplified from unspliced cDNA or genomic DNA. For MHC II and β_2_M, unspliced product was not seen as it would be very large. -RT = control samples without reverse transcription. UI = uninfected. No difference was observed in IE1 or M33 transcription, or in MHC II induction.
**c**. RAW-C2TA cells were infected with GFP^+^ WT or GFP^+^ M78^-^ MCMV (3 p.f.u. / cell, 72h) then flow cytometrically sorted into GFP^+^ and GFP^-^ fractions. RNA was extracted, reverse-transcribed and amplified by PCR as in **b**, using primers for MHC II and β_2_M. nil = no template control. MHC II band intensity is shown, normalised by β_2_M band intensity for the same sample (mean ± SEM of triplicate samples). MHC II induction was evident in the GFP^-^ cells of infected cultures. GFP^+^ cells showed no MHC II transcriptional shut-down.
(PDF)

S2 Fig T cell depletion. Mice were given i.p. every 48h 200μg protein G-purified anti-CD8α (2.43) or anti-CD4 (GK1.5) mAb, starting 96h before infection. Control = no antibody. Spleens taken at 10 days post-infection were analysed for CD4^+^ and CD8^+^ T cells by flow cytometry with antibodies to CD4 (RMA4-4 and CD8β (mAb H35-17.2). Numbers show mean ± SEM of FSC/SSC-gated lymphocytes for 5 mice.
(PDF)
