# Supplementary figures and images for: Murine cytomegalovirus degrades MHC class II to colonize the salivary glands
Source: PLoS Pathog. 2018 Feb 15;14(2):e1006905. doi: 10.1371/journal.ppat.1006905 (PMC5831752; doi:10.1371/journal.ppat.1006905)

Figure S1

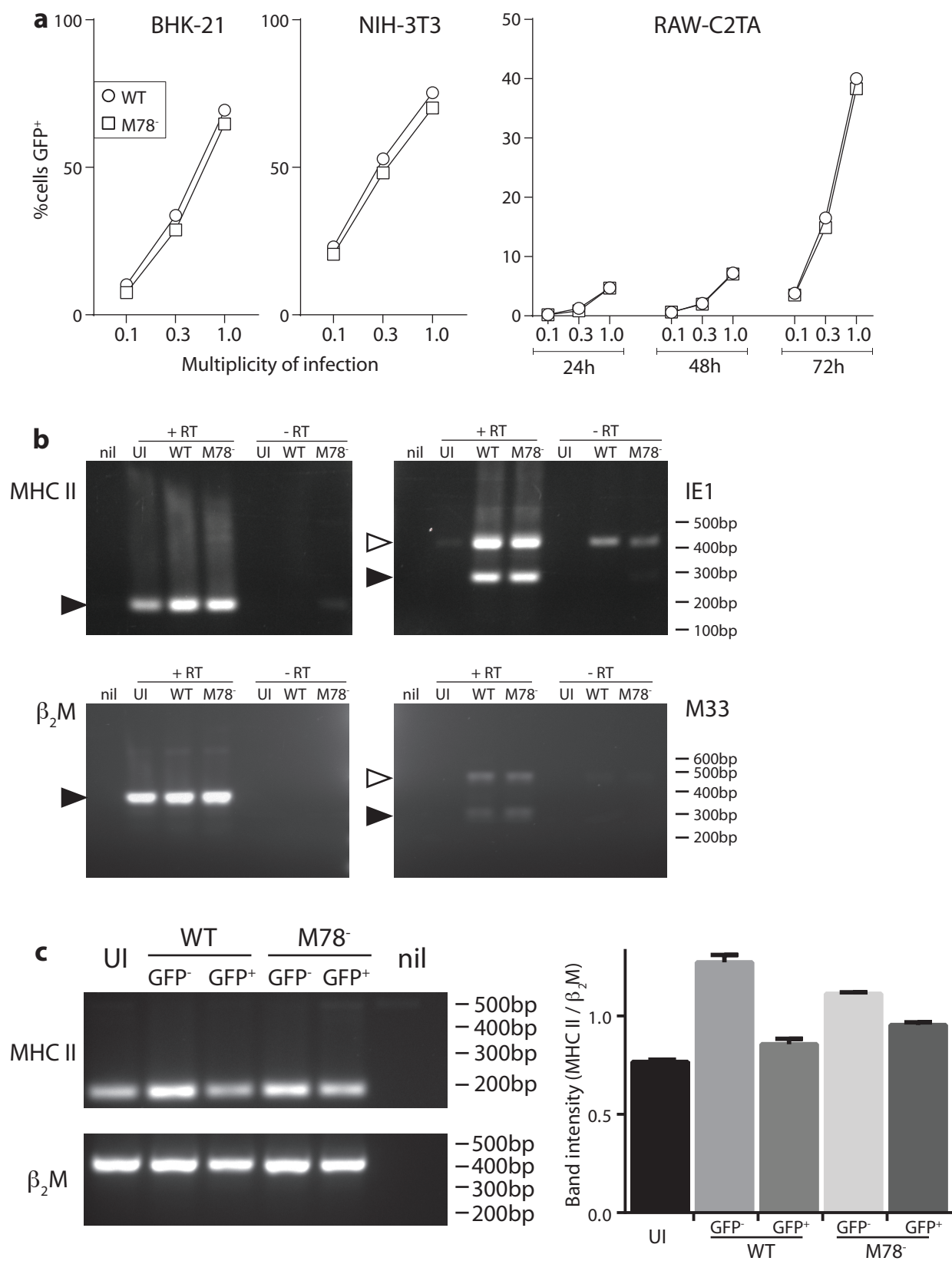

Supplement: S1 Fig — a. BHK-21, NIH-3T3 cells and RAW-C2TA cells were infected with GFP+ WT or GFP+ M78- MCMV at different multiplicities. GFP expression was then quantified by flow cytometry, after 18h for BHK-21 and NIH-3T3 cells, and after 24, 48 and 72 hours for RAW-C2TA cells. No significant difference in infection was observed between WT and M78- MCMV. b. RAW-C2TA cells were infected with WT or M78- MCMV (3 p.f.u. / cell, 72h). RNA was then harvested, reverse transcribed with an oligo-dT primer and used to amplify MHC II, β2-microglobulin (β2M), the MCMV IE1 or the MCMV M33. Each primer set spanned an intron. The filled arrow shows the predicted size of the product amplified from spliced cDNA, and the open arrow that amplified from unspliced cDNA or genomic DNA. For MHC II and β2M, unspliced product was not seen as it would be very large. -RT = control samples without reverse transcription. UI = uninfected. No difference was observed in IE1 or M33 transcription, or in MHC II induction. c. RAW-C2TA cells were infected with GFP+ WT or GFP+ M78- MCMV (3 p.f.u. / cell, 72h) then flow cytometrically sorted into GFP+ and GFP- fractions. RNA was extracted, reverse-transcribed and amplified by PCR as in b, using primers for MHC II and β2M. nil = no template control. MHC II band intensity is shown, normalised by β2M band intensity for the same sample (mean ± SEM of triplicate samples). MHC II induction was evident in the GFP- cells of infected cultures. GFP+ cells showed no MHC II transcriptional shut-down. (PDF) [file ppat.1006905.s001.pdf]

Figure S2

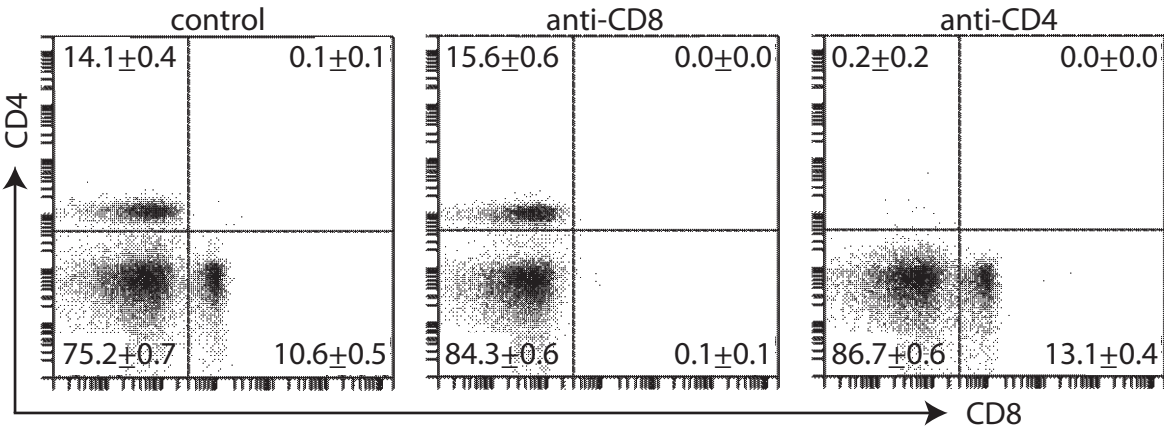

Supplement: S2 Fig — Mice were given i.p. every 48h 200μg protein G-purified anti-CD8α (2.43) or anti-CD4 (GK1.5) mAb, starting 96h before infection. Control = no antibody. Spleens taken at 10 days post-infection were analysed for CD4+ and CD8+ T cells by flow cytometry with antibodies to CD4 (RMA4-4 and CD8β (mAb H35-17.2). Numbers show mean ± SEM of FSC/SSC-gated lymphocytes for 5 mice. (PDF) [file ppat.1006905.s002.pdf]
